# Supplementary material for: Pre- and postsynaptic upregulation of FasII synergistically underlies neuropathological and behavioral phenotypes in a Drosophila model of myotonic dystrophy
Source: Nat Commun. 2025 Dec 18;17:1005. doi: 10.1038/s41467-025-67738-w (PMC12847815; doi:10.1038/s41467-025-67738-w)
Supplement: Supplementary file 1 — Supplementary Information [file 41467_2025_67738_MOESM1_ESM.pdf]

Supplementary Information

Pre- and postsynaptic upregulation of FasII synergistically underlies neuropathological and behavioral phenotypes in a Drosophila model of myotonic dystrophy

Alex Chun Koon[1]*, Ka Yee Winnie Yeung[1]*, Yitao Wu[1], Lok I Leong[1], John Tsun Po Cheung[1], Zhefan Stephen Chen[1,2], Shaohong Isaac Peng[1], Noah S. Armstrong[3], C. Andrew Frank[3], Paul Magneron[4], Mário Gomes-Pereira[4], Joyce Man See Fung[1], Ariadna Bargiela[5,6], Nerea Moreno[5,6,7], Javier Poyatos-Garcia[5,6,7,8], Juan Vilchez[5,7,8], Aline Huguet-Lachon[4], Cassandra Kussius Brewer[9], Max Zinter[9], Erin S. Beck[10], Rubén Artero[5,6,7], Genevieve Gourdon[4], Vivian Budnik[9], Travis Thomson[9], Brian D. McCabe[11] and Ho Yin Edwin Chan[1,2,12†]

Author affiliations:

[1]School of Life Sciences, Faculty of Science, The Chinese University of Hong Kong, Shatin, N.T., Hong Kong SAR, China.

[2]Gerald Choa Neuroscience Institute, The Chinese University of Hong Kong, Shatin, N.T., Hong Kong SAR, China

[3]Interdisciplinary Graduate Program in Neuroscience, Department of Anatomy and Cell Biology, Carver College of Medicine, University of Iowa, Iowa City, Iowa 52242, USA.

[4]Sorbonne Université, Inserm, Institut de Myologie, Centre de Recherche en Myologie, Paris, France.

[5]CIBER de Enfermedades Raras, Instituto de Salud Carlos III, Madrid, Spain.

[6]Incliva Biomedical Research Institute, 46010 Valencia, Spain.

[7]Human Translational Genomics Group, University Institute of Biotechnology and Biomedicine (BIOTECMED), University of Valencia, 46100 Burjassot, Spain.

[8]Neuromuscular and Ataxias Research Group, Health Research Institute Hospital, La Fe (IIS La Fe), 46010, Valencia, Spain.

[9]Department of Neurobiology, University of Massachusetts Chan Medical School, Worcester, MA 01605, USA.

[10]Department of Neurology, Icahn School of Medicine at Mount Sinai, New York, NY, USA.

[11]Brain Mind Institute, EPFL - Swiss Federal Institute of Technology, Lausanne, Switzerland.

[12]State Key Laboratory of Agrobiotechnology (CUHK), The Chinese University of Hong Kong, Shatin, N.T., Hong Kong SAR, China

*These authors contributed equally to this work.

[†]**Corresponding Author:** Ho Yin Edwin Chan, School of Life Sciences, The Chinese University of Hong Kong, Shatin, N.T., Hong Kong SAR, China, Tel: +852-3943-4021; Fax: +852-2603-7732; E-mail address: hyechan@cuhk.edu.hk

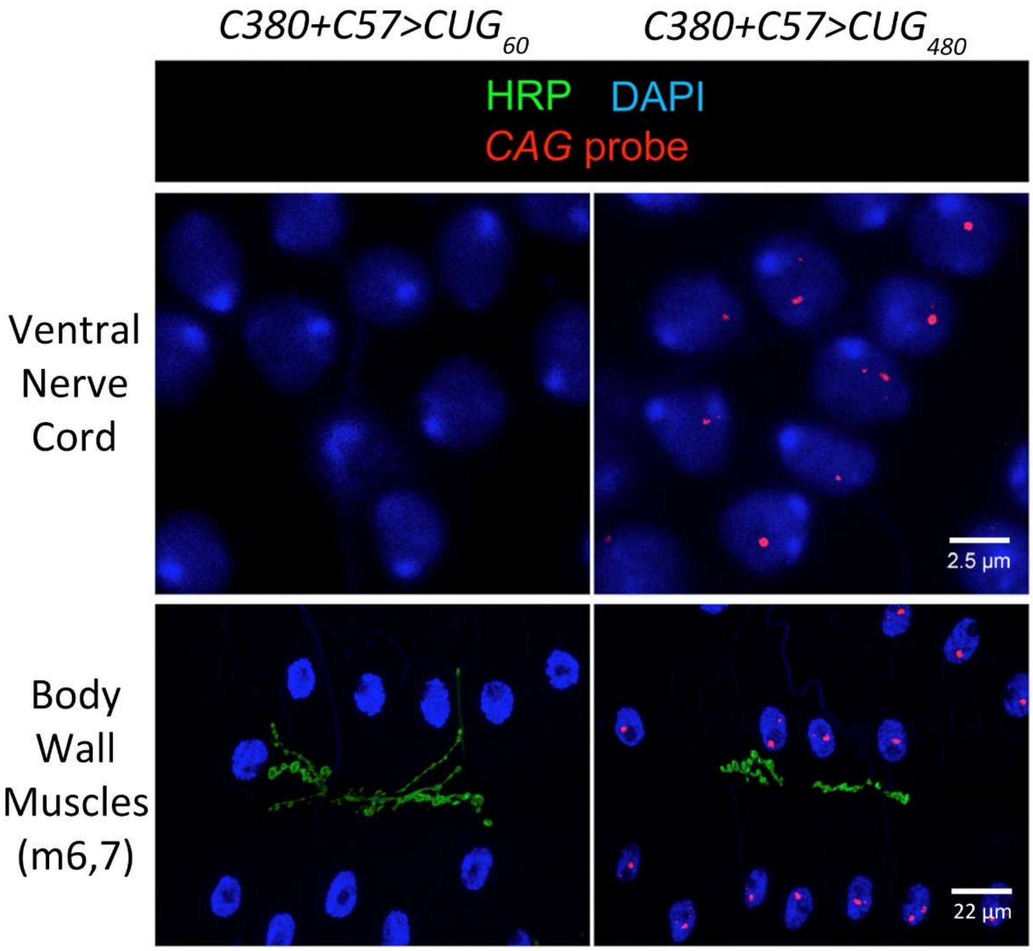

Supplementary Figure 1. *CUG_{480}*-expressing motorneurons and body wall muscles display RNA foci with *CUG* repeats. Fluorescence in situ hybridization using a *CAG* DNA probe that detects *CUG* RNA repeats. Upper panels showing the nuclei of motorneurons residing in the ventral nerve cord of the *Drosophila* larva. Lower panels showing the nuclei of body wall muscles (muscles 6 & 7) of the *Drosophila* larva. Anti-HRP staining (in green) allowed the NMJs to be visible on m6,7.

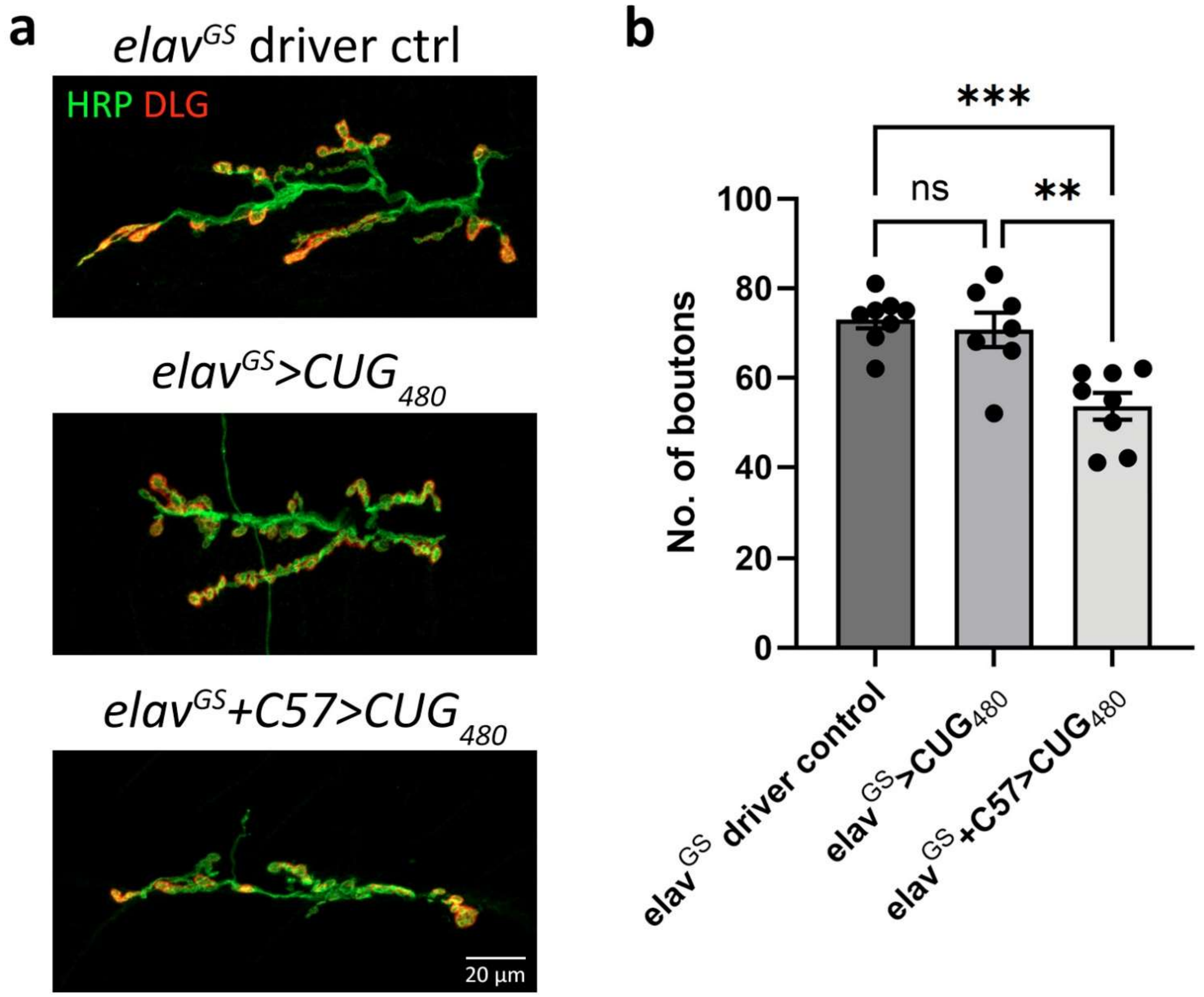

Supplementary Figure 2. Simultaneous pre+postsynaptic expression of *CUG480* using *elav*^{GS}*-Gal4* and *C57-Gal4* causes NMJ phenotypes. (a) Confocal micrographs of *Drosophila* NMJs of late 3rd instar larvae at muscles 6 and 7 of segment A3. Anti-HRP (in green) marks the presynaptic boutons. Anti-Discs large (DLG) (in red) marks the postsynaptic density. Scale bar is 20 µm. **(b)** Quantification of bouton numbers in **(a)**. n = 8, 7, 8, where n is the number of analyzed NMJs. Each larva is defined as a biological replicate, and no more than two NMJs were analyzed per larva. One-way ANOVA with Tukey post-hoc test was performed. Histograms depict mean ± SEM. $^{**}p < 0.01$, $^{***}p < 0.001$.

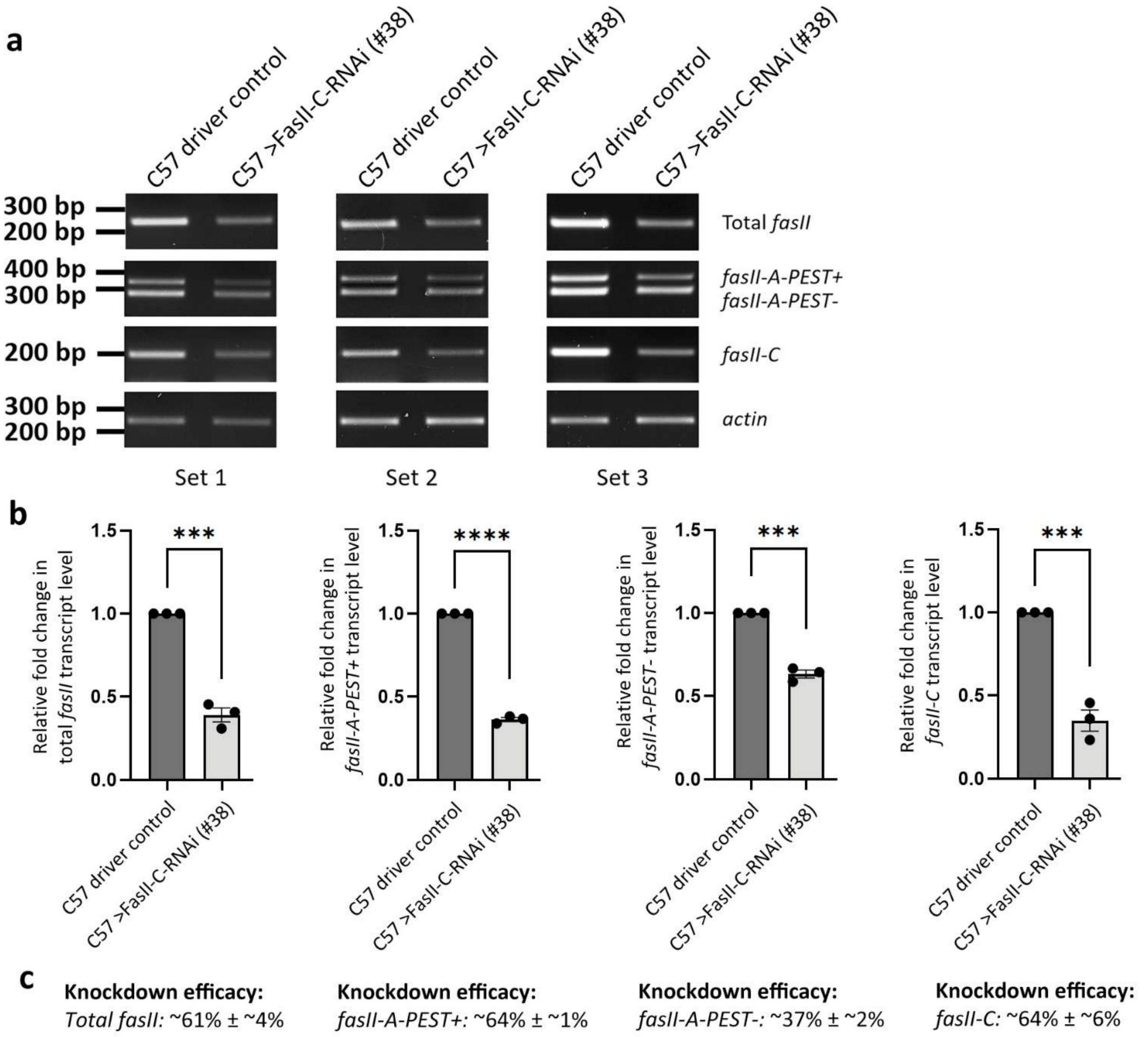

Supplementary Figure 3. ***UAS-FasII-C-RNAi (#38)*** **(which was renamed as *UAS-FasII-A&C-RNAi*) knocks down at least three isoforms of *fasII*.** **(a)** Representative semi-quantitative RT-PCR of total *fasII* and various *fasII* isoforms in *Drosophila* larval body wall muscles (BWMs). *C57-Gal4* is a BWM driver. **(b)** Quantification of **(a)**. **(c)** Knockdown efficacies of total *fasII* and various *fasII* isoforms. N = 3. Each N is an independent experiment and is defined as a biological replicate. Student's t-test (two-tailed) was used. Histograms depict mean ± SEM. ***$p < 0.001$, ****$p < 0.0001$.

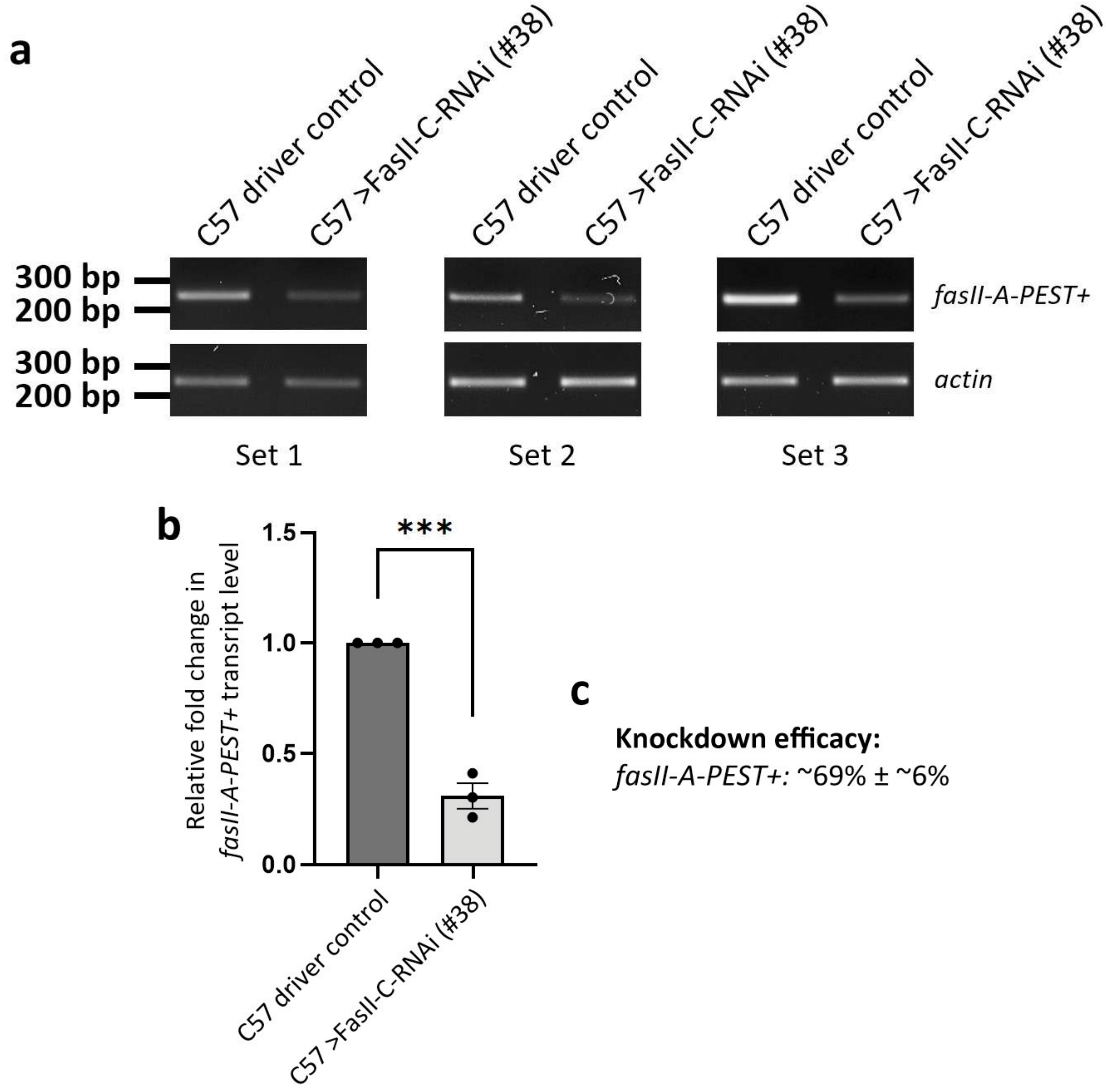

Supplementary Figure 4. ***UAS-FasII-C (#38)*** **knocks down the PEST+ isoform of** ***fasII.*** **(a)** Representative semi-quantitative RT-PCR of *fasII-A-PEST+* in *Drosophila* larval BWMs. The forward primer used in this PCR targets the nucleotide sequence of Exon 7 and the transmembrane domain, while the reverse primer targets the nucleotide sequence of the PEST domain. Hence, these primers exclusively detect the *fasII-A-PEST+* isoform. **(b)** Quantification of **(a)**. **(c)** Knockdown efficacy of *fasII-A-PEST+*. N = 3. Each N is an independent experiment and is defined as a biological replicate. Student's t-test (two-tailed) was used. Histograms depict mean ± SEM. ***p < 0.001.

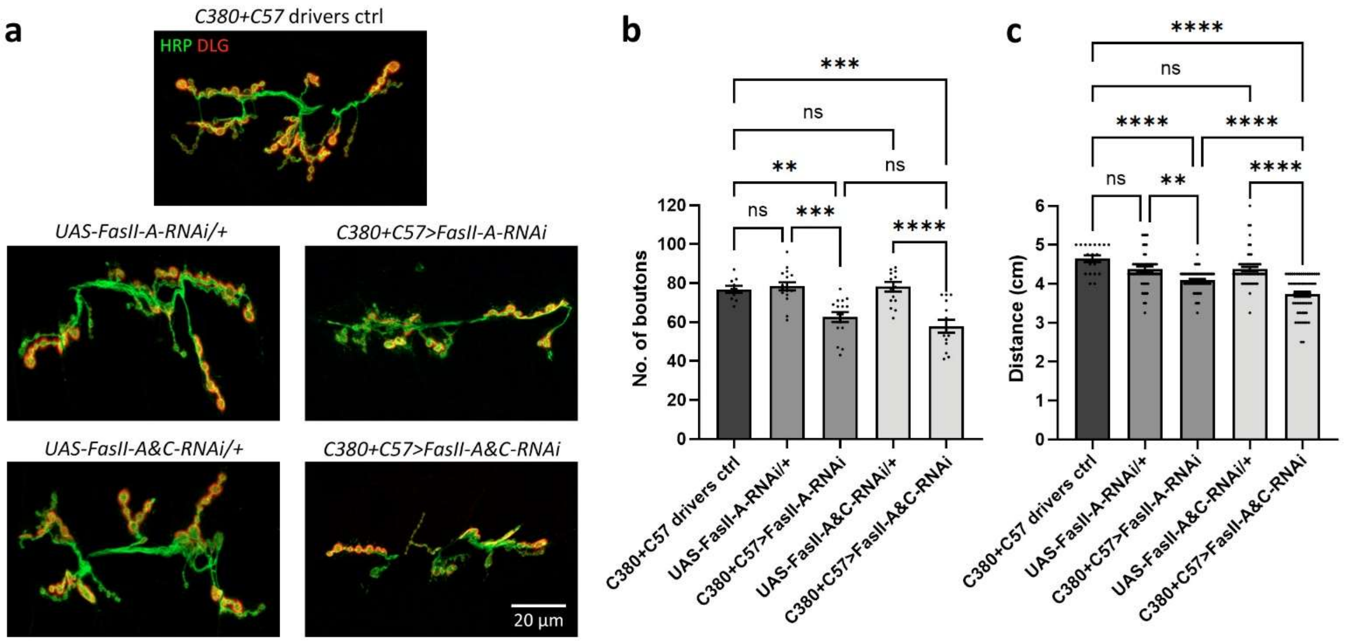

Supplementary Figure 5. Knockdown of FasII-A using *UAS-FasII-A-RNAi* or FasII-A & C using *UAS-FasII-A&C-RNAi* at the NMJ results in reduction of boutons. (a) Confocal micrographs of *Drosophila* NMJs of late 3rd instar larvae at muscles 6 and 7 of segment A3. Anti-HRP (in green) marks the presynaptic boutons. Anti-Discs large (DLG) (in red) marks the postsynaptic density. Scale bar is 20 µm. **(b)** Quantification of bouton numbers in **(a)**. n = 10, 17, 16, 15, 14, where n is the number of analyzed NMJs. **(c)** Quantification of larval locomotor activity. n = 60, 60, 60, 60, 60, 60, where n indicates the number of analyzed larvae. Each larva is defined as a biological replicate, and no more than two NMJs were analyzed per larva. One-way ANOVA with Tukey post-hoc test was performed. Histograms depict mean ± SEM. $^{**}p < 0.01$, $^{***}p < 0.001$, $^{****}p < 0.0001$.

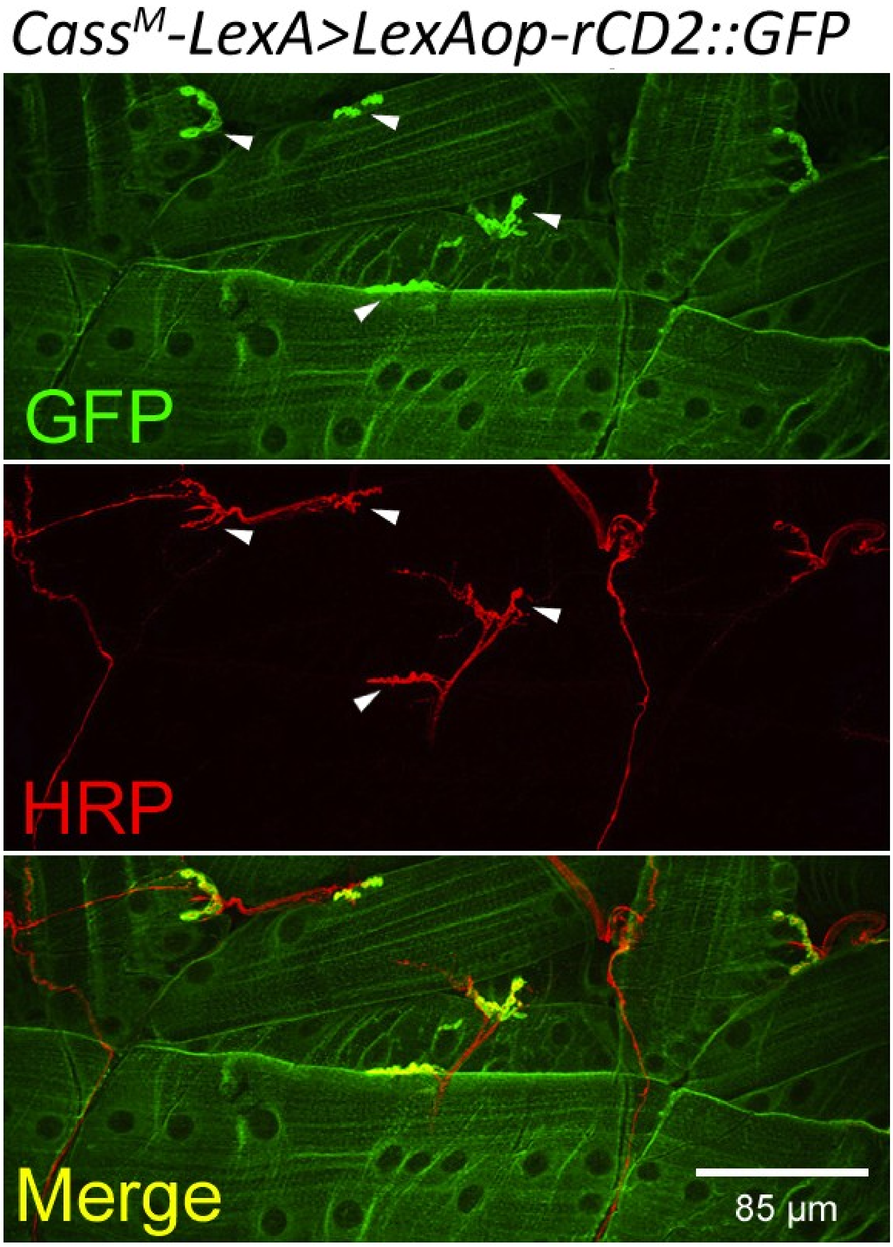

Supplementary Figure 6. *Cass^{M}-LexA* expresses in body wall muscles but not in motor neurons at the *Drosophila* larval NMJ. Confocal micrograph of *Drosophila* NMJs of late 3rd instar larvae at muscle 5, 6, 8, 12 and 13 of segment A3. Anti-GFP (in green) marks the membrane of the body wall muscles. Anti-HRP (in red) marks the motor neurons. White arrowheads denote regions of synaptic boutons. On the muscles, the membrane-rich subsynaptic reticulum (SSR) surrounds the boutons. Thus, the membrane-tethered rCD2::GFP were observed at the SSR in the shape of boutons, even though there was no expression of GFP in the presynaptic boutons. Scale bar is 85 µm.

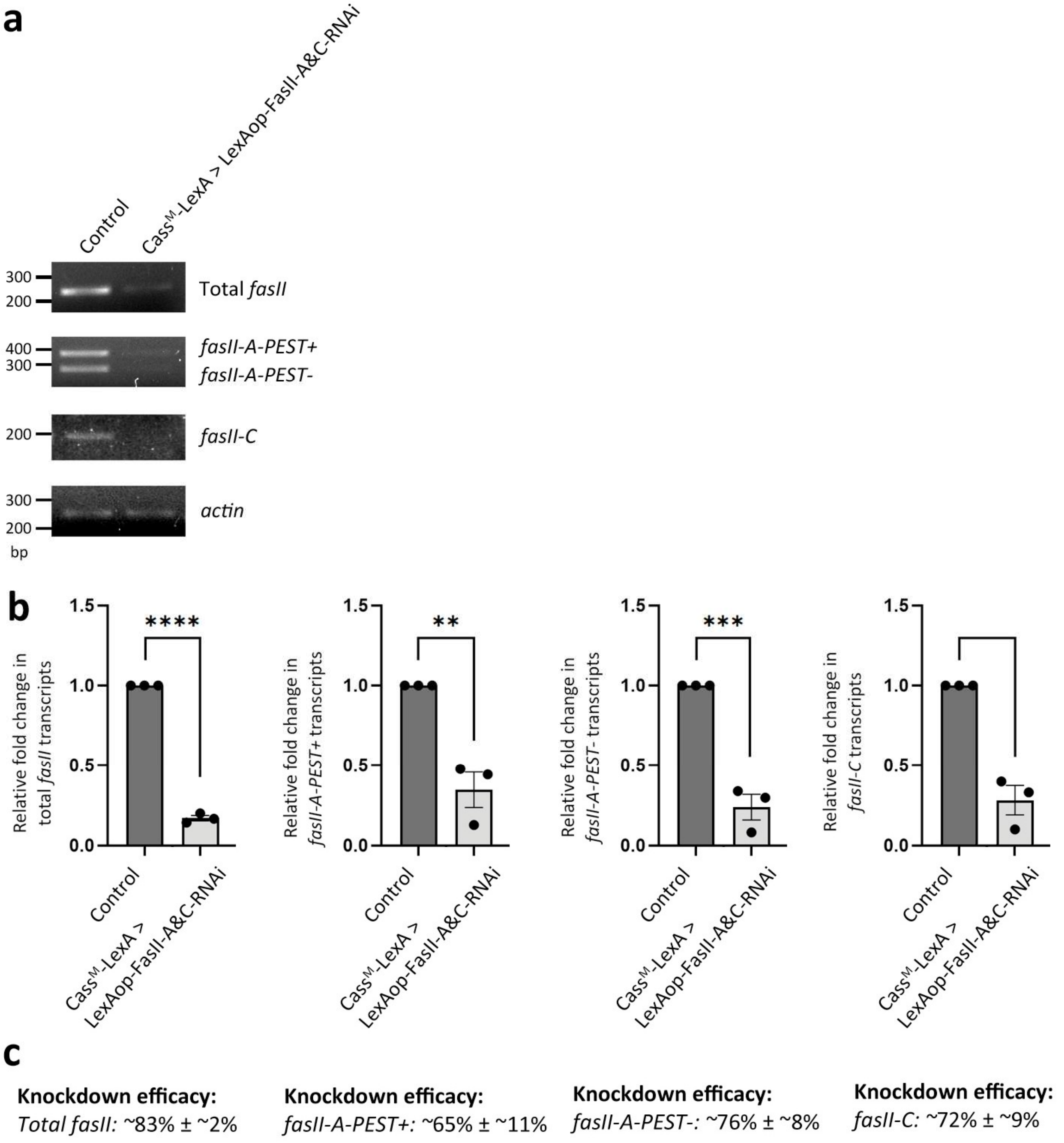

Supplementary Figure 7. *LexAop-FasII-A&C-RNAi* knocks down the *fasII-A* and *fasII-C* isoforms. **(a)** Representative semi-quantitative RT-PCR of total *fasII* and various *fasII* isoforms in *Drosophila* larval body wall muscles. **(b)** Quantification of **(a)**. **(c)** Knockdown efficacies of total *fasII* and various *fasII* isoforms. N = 3. Each N is an independent experiment and is defined as a biological replicate. Student's t-test (two-tailed) was used. Histograms depict mean ± SEM. ***p < 0.001, ****p < 0.0001.

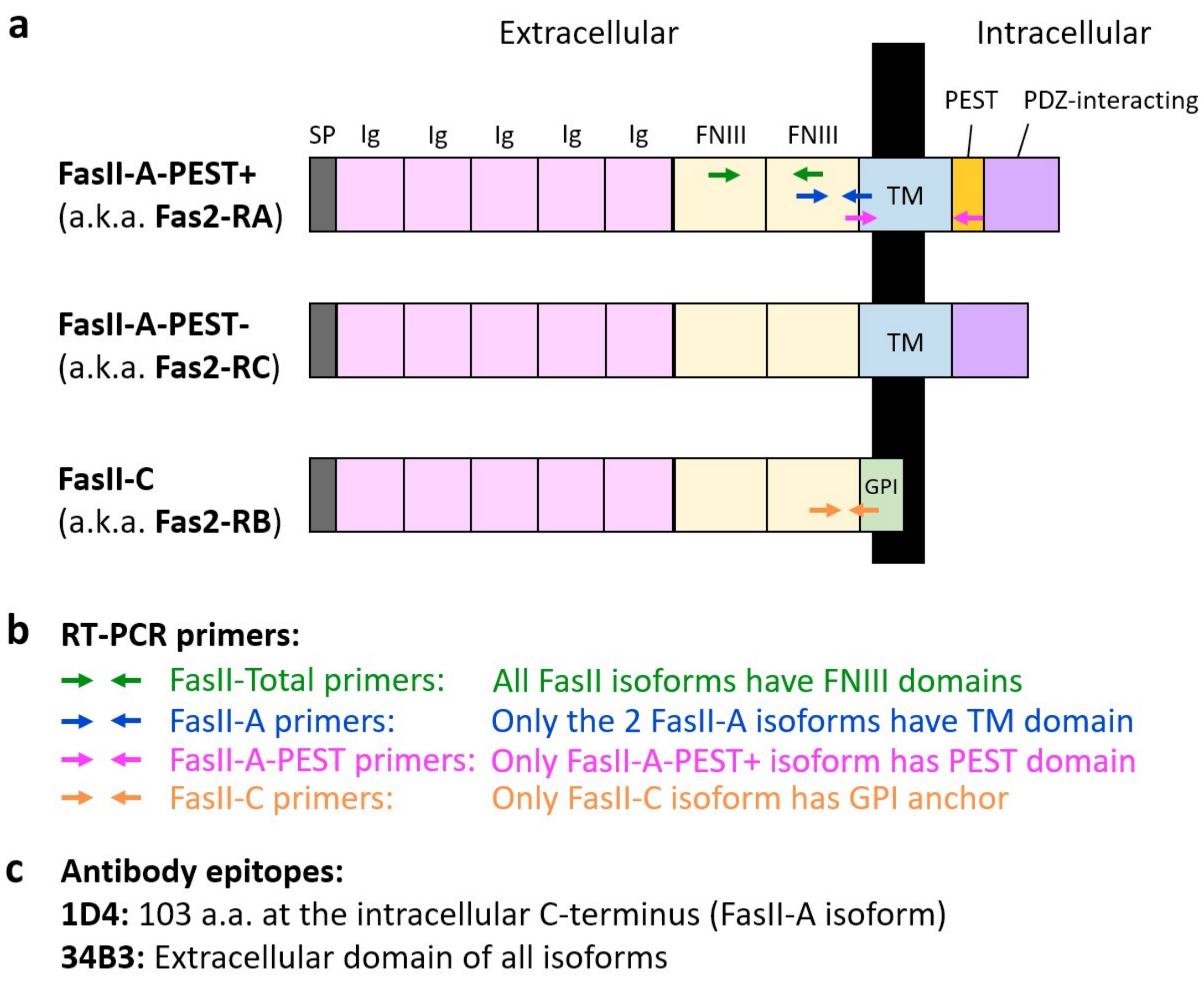

Supplementary Figure 8. FasII isoforms and their functional domains. (a) Schematic representation of the characterized FasII isoforms and their functional domains. SP: signal peptide; Ig: immunoglobulin-like (Ig-like) domain; FNIII: fibronectin type III domain; TM: transmembrane domain; PEST: PEST domain; GPI: GPI anchor. **(b)** Colored arrows denote RT-PCR primers designed to amplify specific FasII isoform(s). **(c)** List of epitopes recognized by anti-FasII antibodies. There are two available *Drosophila* FasII antibodies that have been previously characterized: 1D4 and 34B3. 1D4 can only detect the two FasII-A isoforms [68], while 34B3 can potentially detect all FasII isoforms [41].

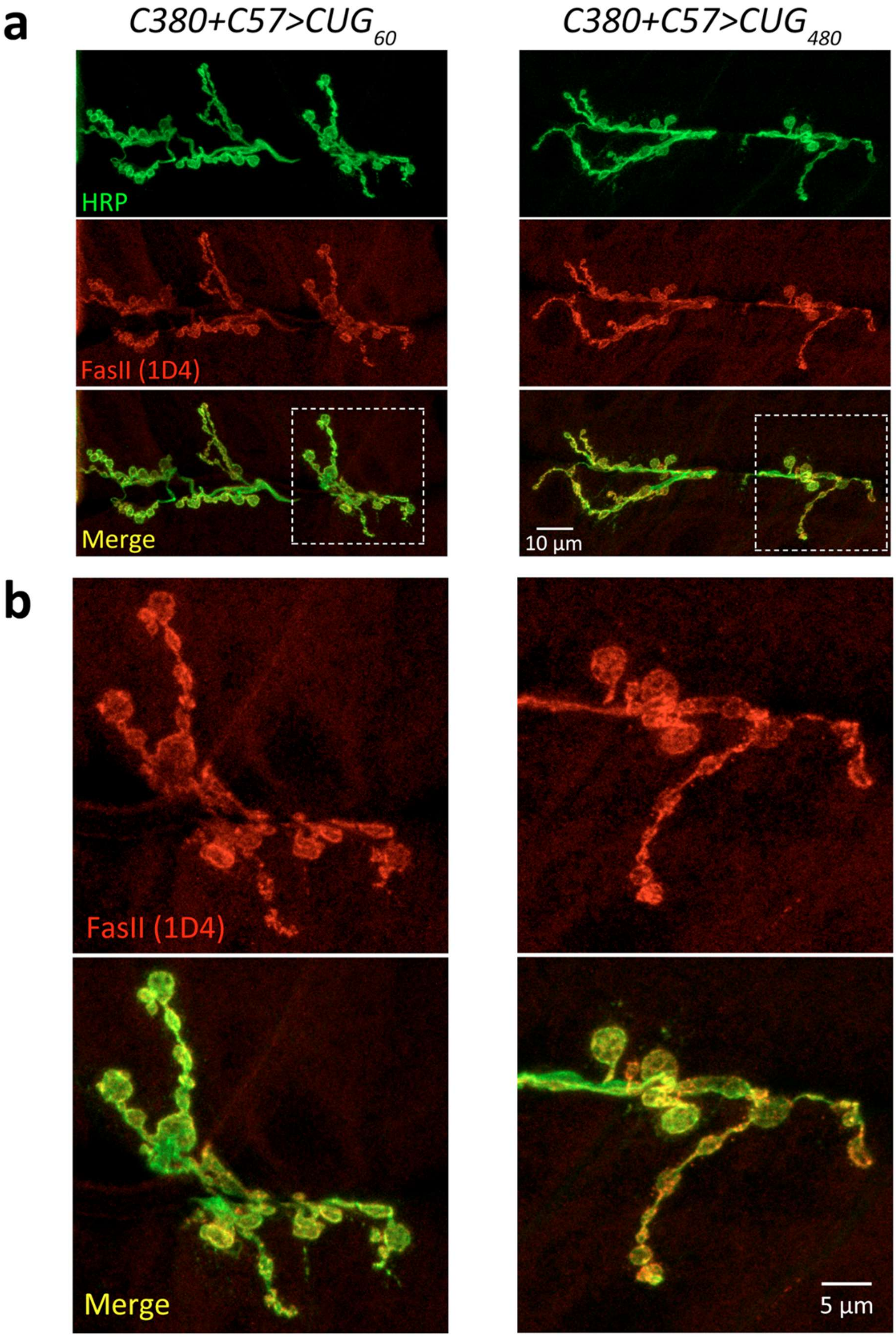

Supplementary Figure 9. No changes in spatial distribution of FasII-A were detected at the NMJs of *CUG480*-expressing larvae. (a) Confocal micrographs of *Drosophila* NMJs of late 3rd instar larvae at muscles 6 and 7 of segment A3. Anti-HRP (in green) marks the presynaptic boutons. Anti-FasII (1D4) is in red. Scale bar is 10 µm. **(b)** High-magnification confocal micrographs of the white dotted squared in **(a)**. Scale bar is 5 µm.

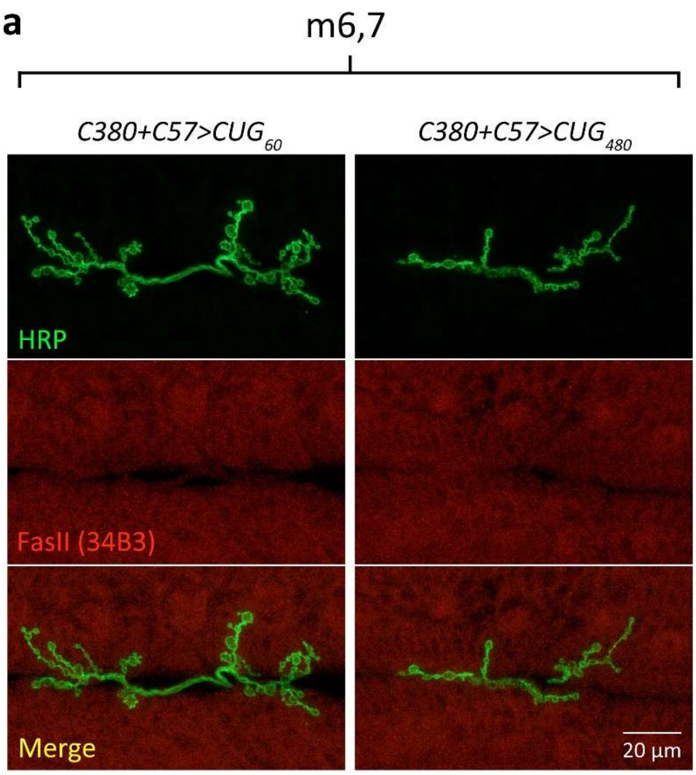

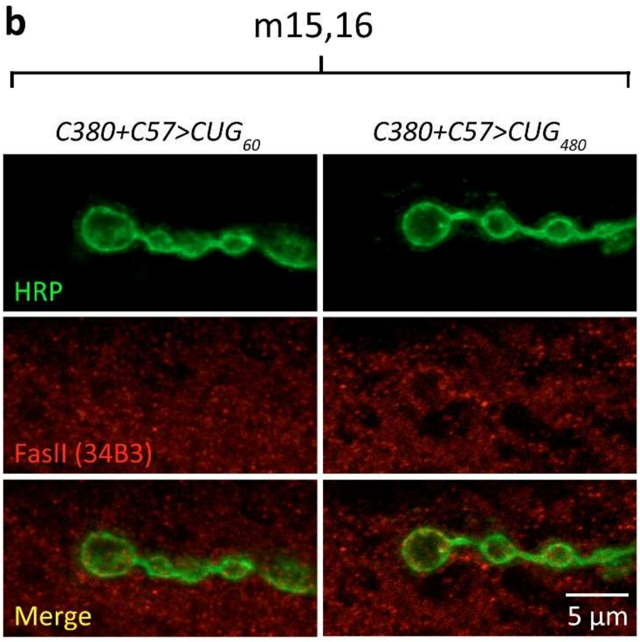

c

	C380+C57>CUG$_{60}$	*C380+C57>CUG*$_{480}$
Percentage of NMJs on m15,16 with 34B3 staining:	0% (*n* = 8)	37.5% (*n* = 8)

Supplementary Figure 10. Nearly no immunostaining on boutons at the larval NMJs could be achieved using anti-FasII (34B3). (a) Confocal micrographs of *Drosophila* NMJs of late 3rd instar larvae at muscles 6 and 7 (m6,7) of segment A3. No visible immunostaining was achieved using 34B3. **(b)** High-magnification confocal micrographs of *Drosophila* NMJs of late 3rd instar larvae at muscles 15 and 16 (m15,16) of segment A5. Barely visible immunostaining of FasII using 34B3 was detected in the terminal bouton of *CUG*$_{480}$-expressing animals. Anti-HRP (in green) marks the presynaptic boutons. Anti-FasII (34B3) is in red. Scale bar is 10 µm. **(c)** Quantification of NMJs on m15,16 of segment A5/A6 with FasII signals detected in the terminal bouton using 34B3.

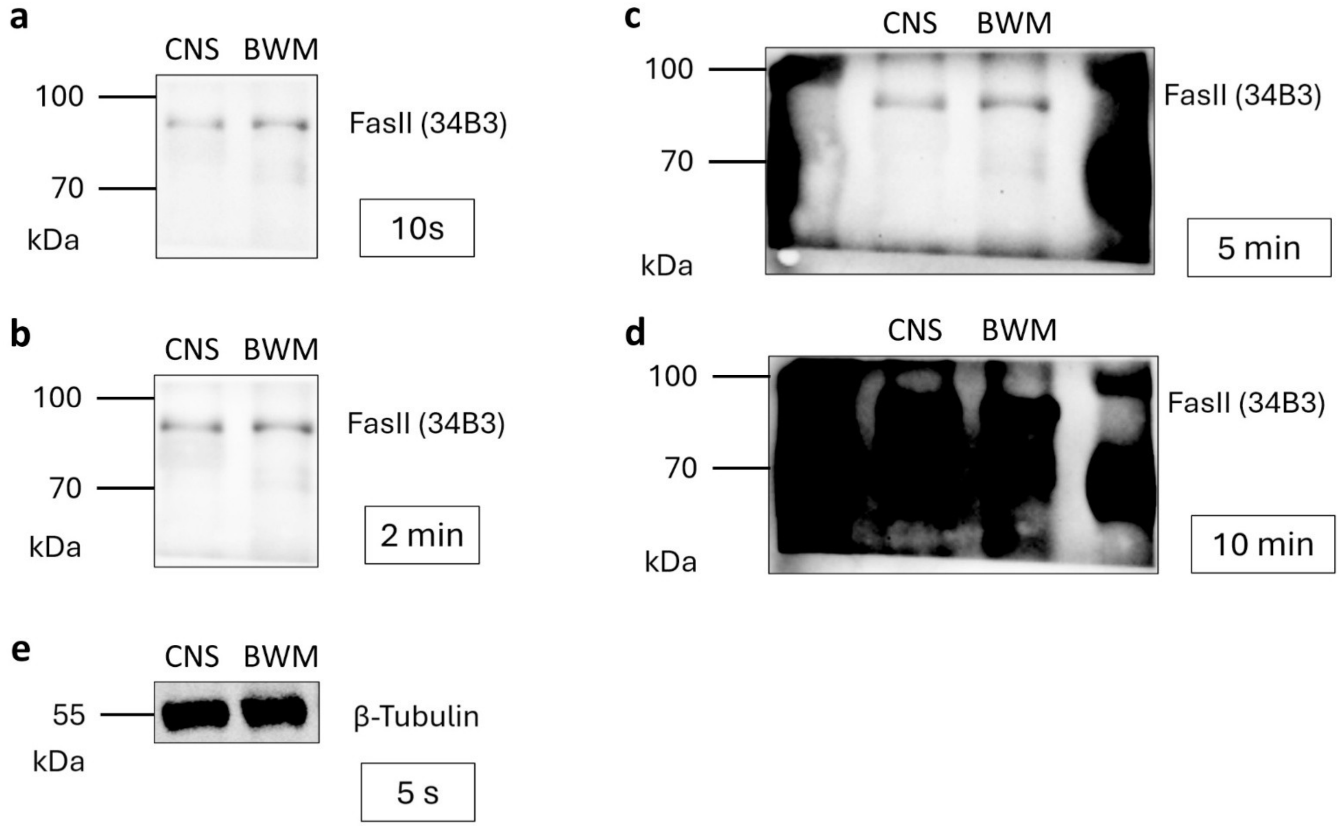

Supplementary Figure 11. Only a single FasII isoform was effectively detected using anti-FasII (34B3) in Western blot. Western blot of FasII in *Drosophila* CNS and BWM using anti-FasII (34B3). The possible band sizes for the different FasII isoforms are: FasII-A-PEST+ (96 kDa), FasII-A-PEST- (93 kDa), FasII-C (90 kDa), FasII-B (86 kDa). Only a band of approximately 96 kDa was detected. **(a)** 10 sec exposure. **(b)** 2 min exposure. **(c)** 5 min exposure. **(d)** 10 min exposure. **(e)** Internal control using anti-β-Tubulin.

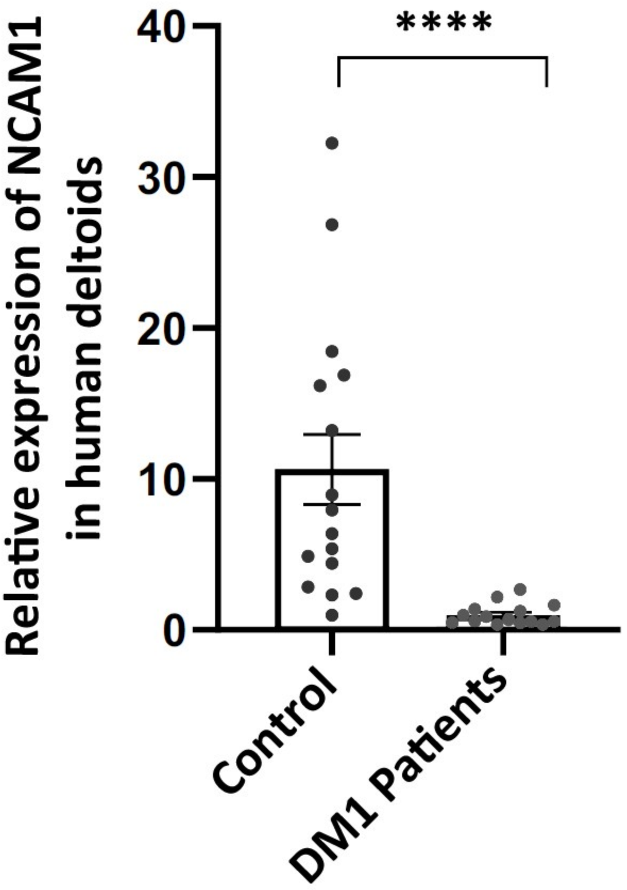

Supplementary Figure 12. Lower expression of NCAM1 was detected in the deltoids of DM1 patients. Quantitative dot blot data of NCAM1 in human deltoids. n = 16,15. Each n is a biological replicate. Histograms depict mean ± SEM. ****p < 0.0001.

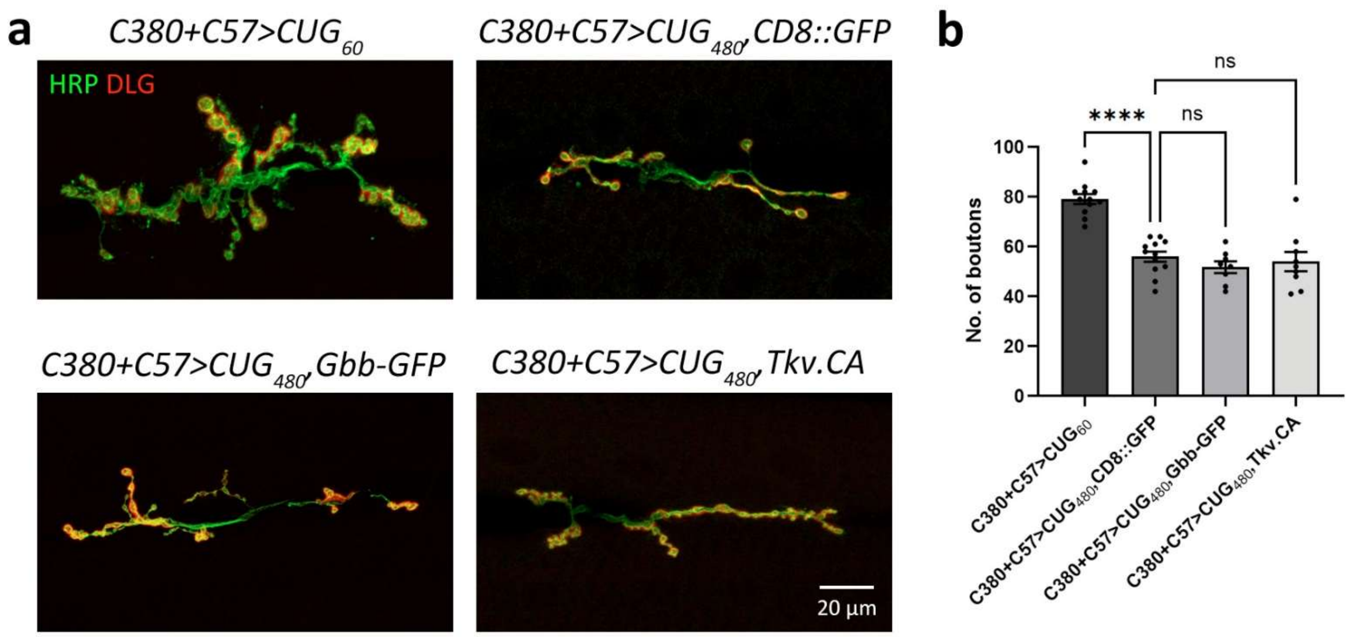

Supplementary Figure 13. Overexpression of Gbb or a constitutively active form of Tkv were unable to rescue the *Drosophila* DM1 model. (a) Confocal micrographs of *Drosophila* NMJs of late 3rd instar larvae at muscles 6 and 7 of segment A3. Anti-HRP (in green) marks the presynaptic boutons. Anti-Discs large (DLG) (in red) marks the postsynaptic density. Scale bar is 20 µm. **(b)** Quantification of bouton numbers in **(a)**. n = 12, 12, 8, 9, where n is the number of analyzed NMJs. Each larva is defined as a biological replicate, and no more than two NMJs were analyzed per larva. One-way ANOVA with Tukey post-hoc test was performed. Histograms depict mean ± SEM. ****$p < 0.0001$.

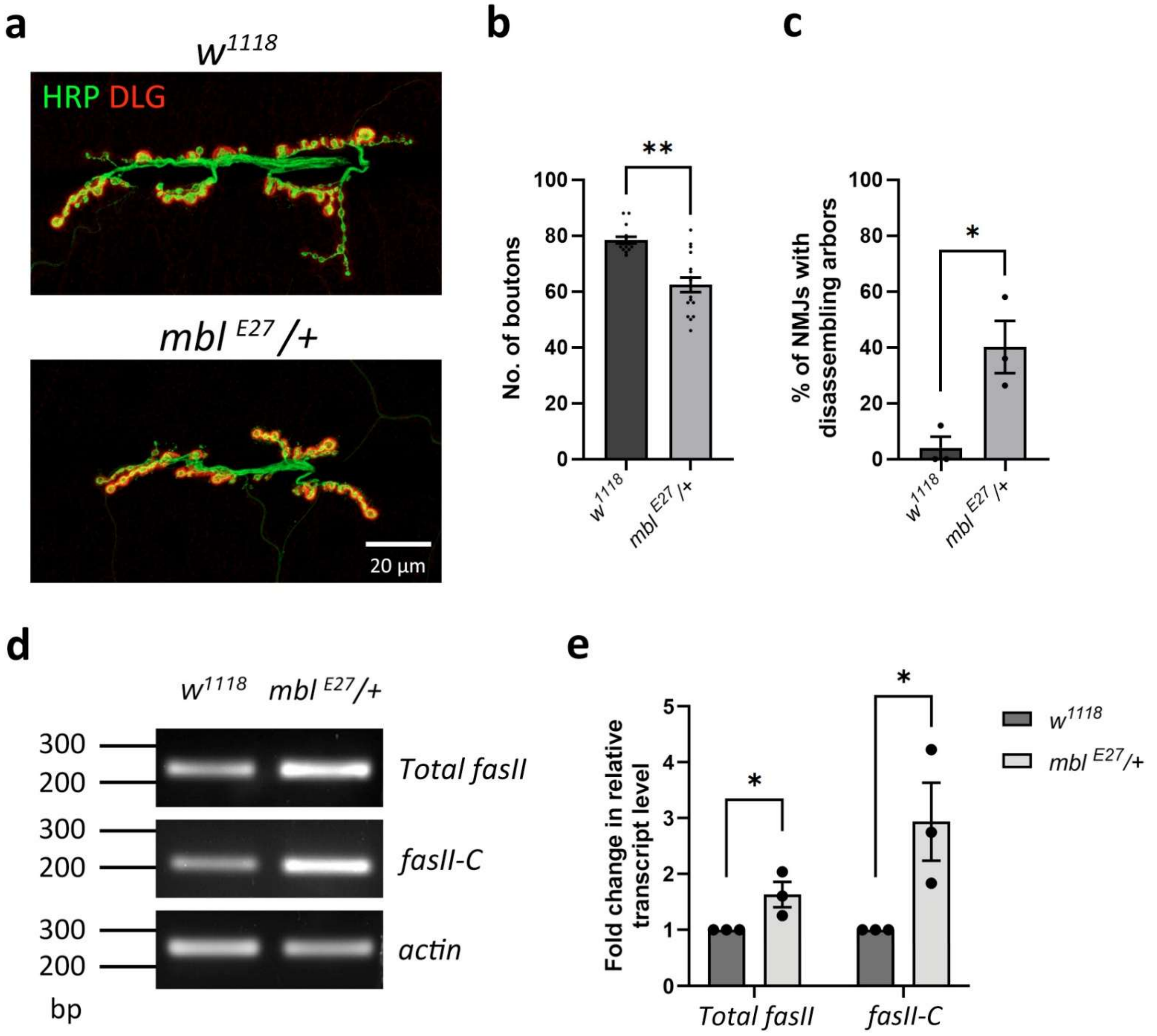

Supplementary Figure 14. Heterozygous *mbl* ^{E27} mutants have phenotypes resembling the *Drosophila* DM1 model. (a) Confocal micrographs of *Drosophila* NMJs of late 3rd instar larvae at muscles 6 and 7 of segment A3. Anti-HRP (in green) marks the presynaptic boutons. Anti-Discs large (DLG) (in red) marks the postsynaptic density. Scale bar is 20 µm. **(b)** Quantification of bouton numbers in **(a)**. n = 15, 15, 15, where n is the number of analyzed NMJs. **(c)** Quantification of disassembling arbors. n = 15, 15, 15, where n is the number of analyzed NMJs. Each larva is defined as a biological replicate, and no more than two NMJs were analyzed per larva. Analysis of variance (one-way ANOVA) with Tukey post-hoc test was performed. **(d)** Representative semi-quantitative RT-PCR of total *fasII* and the *fasII-C* isoform in *Drosophila* larval body wall muscles. **(e)** Quantification of **(d)**. N = 3. Each N is an independent experiment and is defined as a biological replicate. Histograms depict mean ± SEM. *$p < 0.05$, **$p < 0.01$.

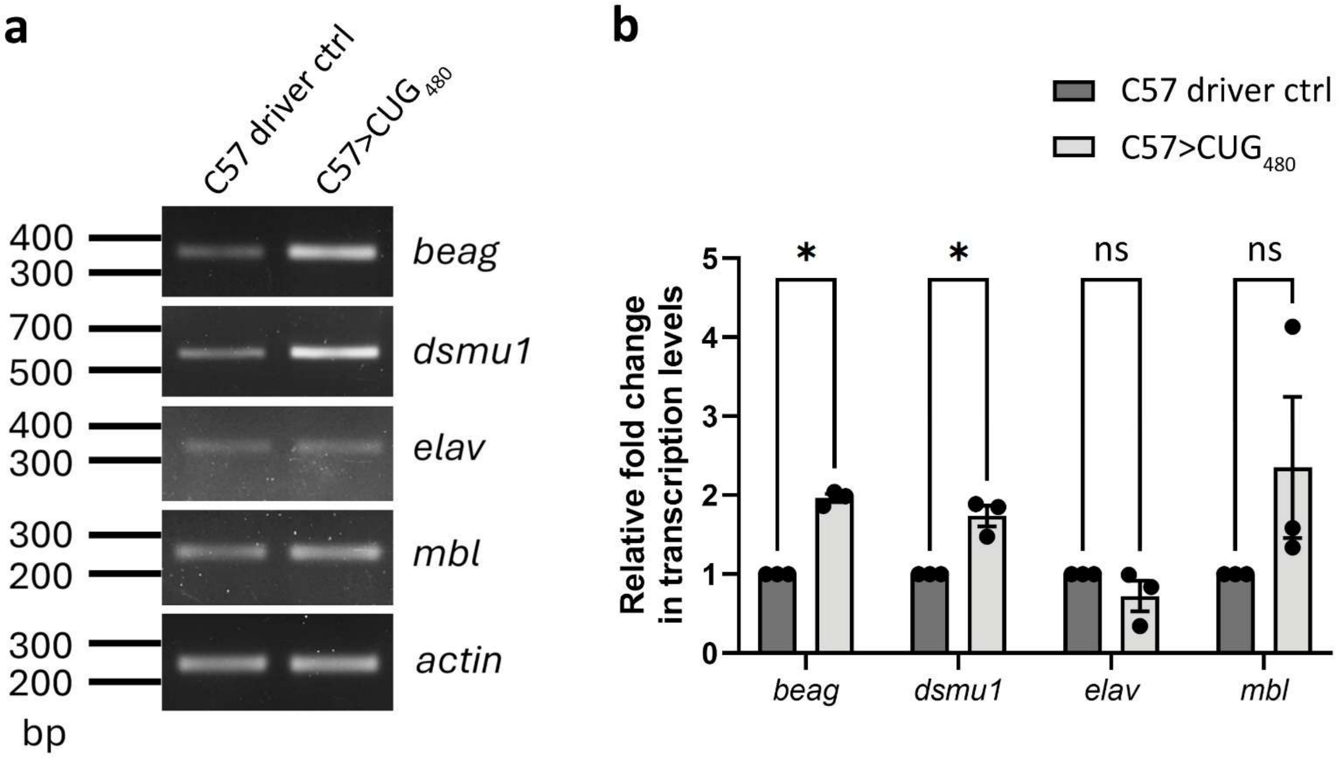

Supplementary Figure 15. Overexpression of *CUG_{480}* results in increased transcription of *beag* and *dsmu1*. **(a)** Representative semi-quantitative RT-PCR of *beag, dsmu1, mbl* and *elav* in *Drosophila* larval body wall muscles overexpressing *CUG_{480}* using *C57-GAL4*. **(b)** Quantification of **(a)**. N = 3. Each N is an independent experiment and is defined as a biological replicate. Histograms depict mean ± SEM. **$p < 0.01$.

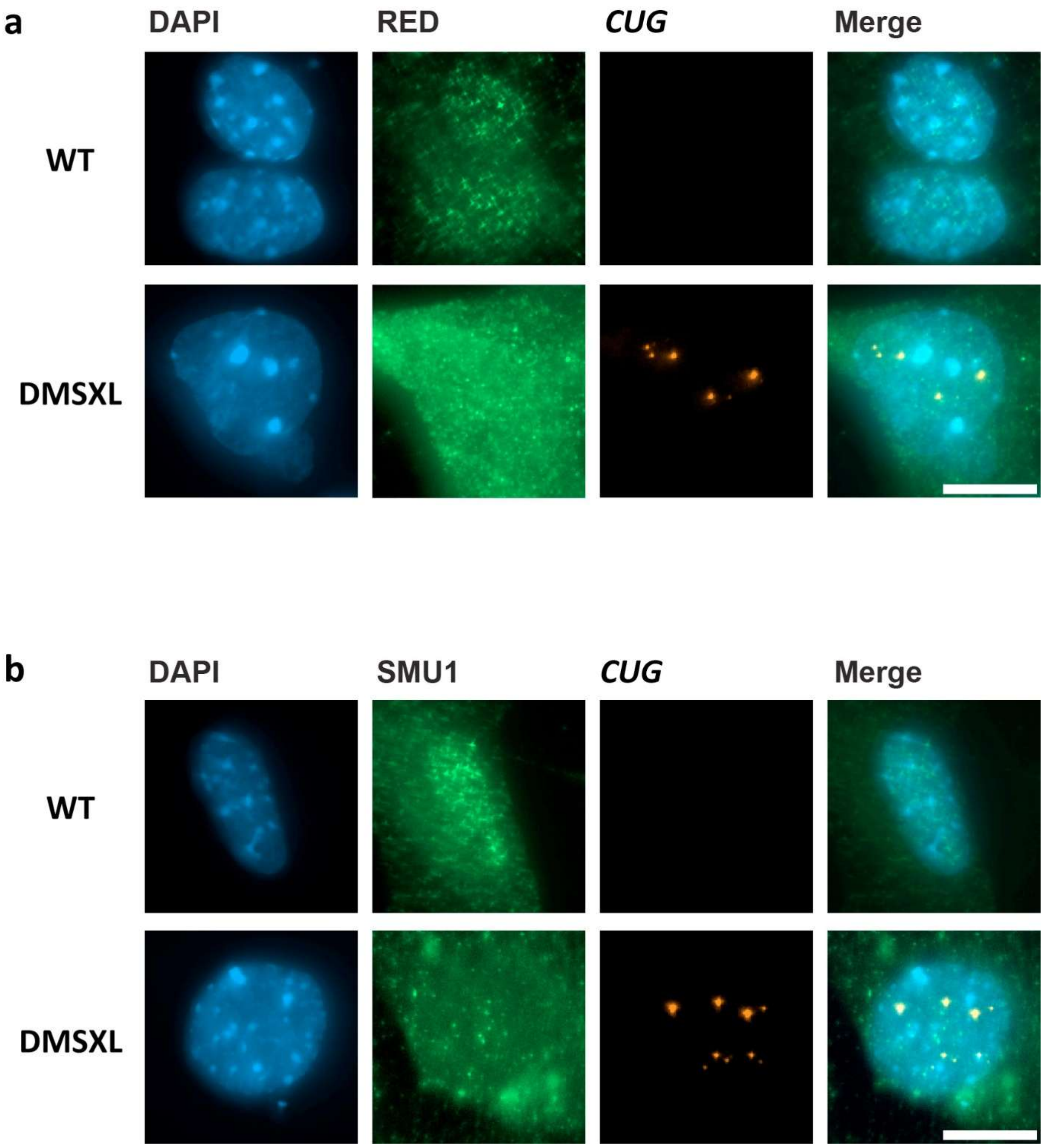

Supplementary Figure 16. *CUG* foci do not sequester RED and SMU1 in primary DMSXL astrocytes. Foci accumulation in cultured mouse astrocytes was detected by FISH, in combination with immunofluorescence of RED **(a)** and SMU1 **(b)**. Nuclei were stained with DAPI. Scale bars represent 10 μm.

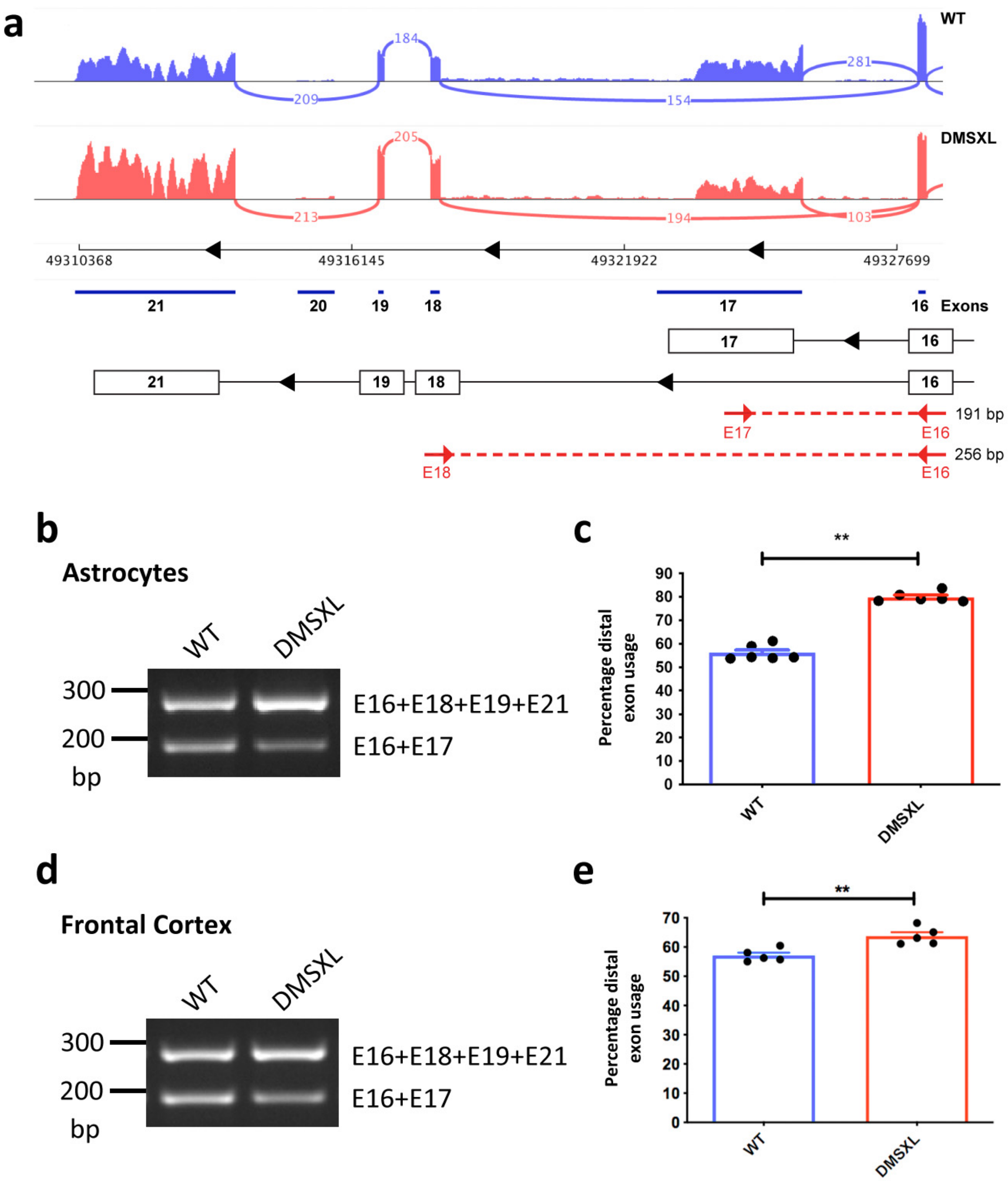

Supplementary Figure 17. Splicing analysis of *Ncam1* transcripts revealed dysregulation in DMSXL mice. **(a)** Representative Sashimi plots of alternative splicing of mouse *Ncam1* RNA in control (WT) and DMSXL primary astrocytes. Exon-exon junction reads (arcs, with numbers indicating supporting read counts) are shown across the genomic region spanning exons 16-21. The main isoforms detected are represented below: control samples (blue) display equivalent inclusion of distal exons 17 and 21, whereas DMSXL astrocytes (red) exhibit increased exon 18-19-21 inclusion. The mapping of oligonucleotide primers used for RT-PCR splicing analysis are indicated in exon 16 (forward) and in exon 17 or 18 (reverse). **(b)** Representative RT-PCR splicing analysis of *Ncam1* transcripts in WT and DMSXL primary astrocytes. **(c)** Quantification of the *Ncam1* RNA isoform containing the distal exons E18, E19 and E21 in **(b)** (n = 6, with each n being an independent culture). **(d)** Representative RT-PCR splicing analysis of *Ncam1* transcripts in the frontal cortex of 1-month-old WT and DMSXL mice. **(e)** Quantification of the *Ncam1* RNA isoform containing the distal exons E18, E19 and E21 in **(d)** (n = 5, n represents mice per genotype). Data are means ± SEM. $^{**}p < 0.01$, Two-tailed Student's t test.

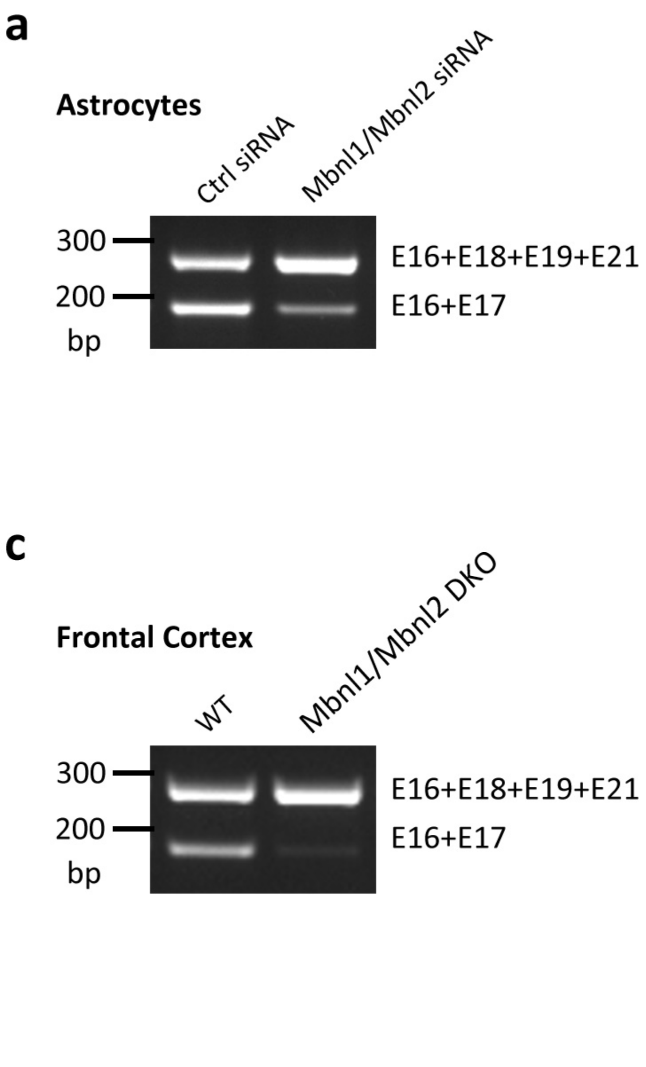

Supplementary Figure 18. Knockdown or conditional double-knockout of Mbnl1/Mbnl2 causes splicing dysregulation of *Ncam1* transcripts. (a) Representative RT-PCR splicing analysis of *Ncam1* transcripts in primary astrocytes. **(b)** Quantification of the *Ncam1* RNA isoform containing the distal exons E18, E19 and E21 in **(a)** (n = 3, with each n being an independent culture). **(c)** Representative RT-PCR splicing analysis of *Ncam1* transcripts in the frontal cortex of P30 WT and *Mbnl1*$^{-/-}$; *Mbnl2*$^{c/c}$; Nestin-Cre+ conditional double-knockout mice (Mbnl1/Mbnl2 DKO). **(d)** Quantification of the *Ncam1* RNA isoform containing the distal exons E18, E19 and E21 in **(c)** (n = 5 and 3, n represents mice per genotype). Data are means ± SEM. ****$p < 0.0001$, Two-tailed Student's t test.

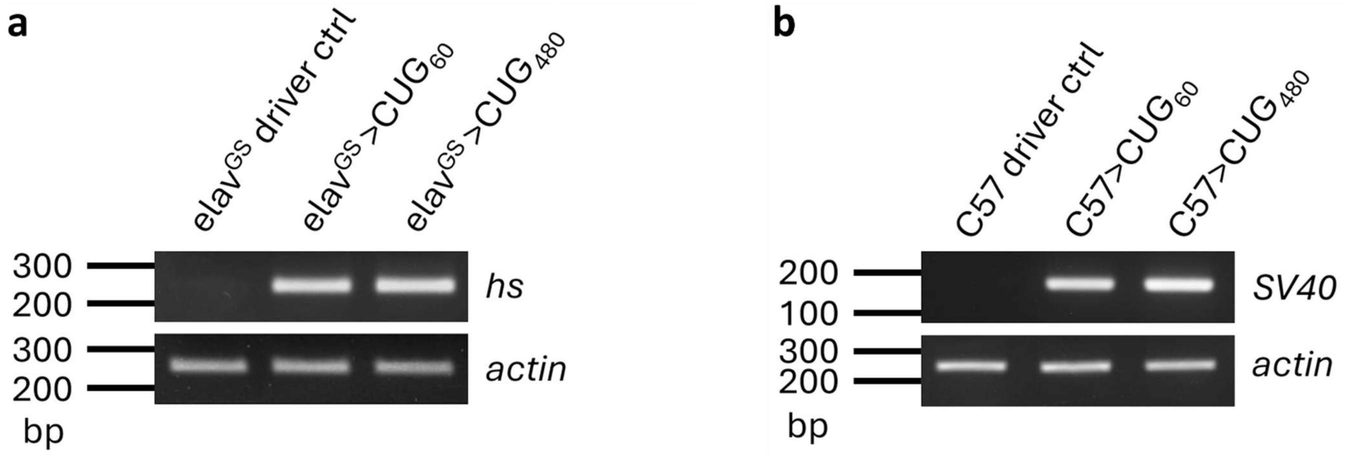

Supplementary Figure 19. Transgene expression levels of *UAS-CTG$_{60}$* and *UAS-CTG$_{480}$* in the *Drosophila* larva. RT-PCR to confirm expression levels of untranslated *UAS-CTG$_{60}$* and *UAS-CTG$_{480}$* constructs in *Drosophila* late 3rd instar larval tissues. Tissue samples were either **(a)** larval CNS of animals expressing *CUG$_{480}$* using the panneuronal *elav^{GS}-GAL4*; or **(b)** body wall muscles of animals expressing *CUG$_{480}$* using *C57-GAL4*. The *heat shock promoter* (*hs*) and *simian vacuolating virus 40* (*SV40*) sequences are presented in the expressed transcripts of the *UAS-CTG$_{60}$* and *UAS-CTG$_{480}$* constructs. The *hs* primers were used to detect the *CUG* transgene expression in the CNS tissues because *hs* was absent in *elav^{GS}-GAL4*, while the *SV40* primers were used to detect the *CUG* transgene expression in the muscle tissues, because *SV40* was absent in *C57-GAL4*.

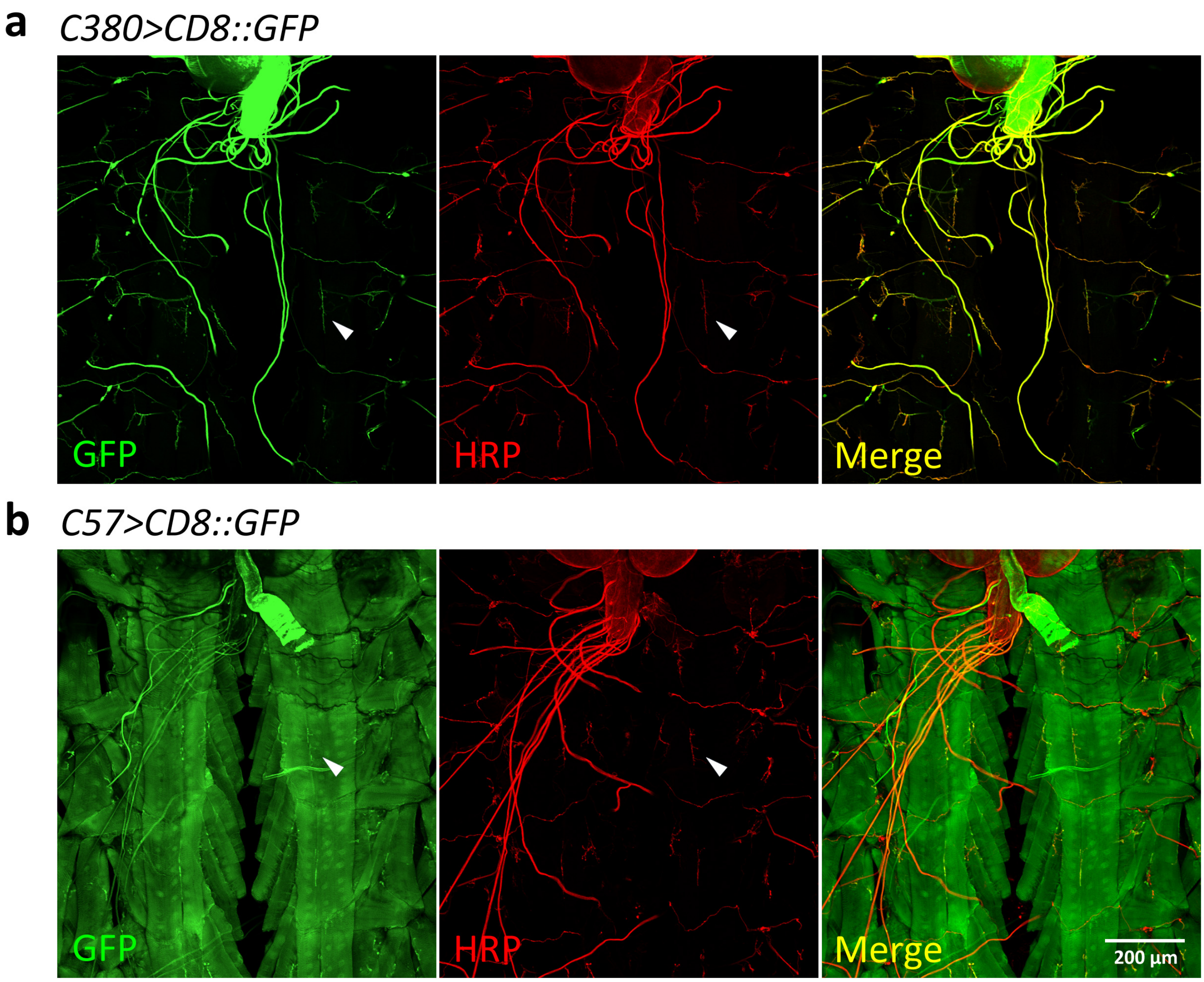

Supplementary Figure 20. Expression patterns of *C380-GAL4* and *C57-GAL4* in the *Drosophila* larva. Confocal micrographs of *Drosophila* late 3rd instar larvae. **(a)** Presynaptic motorneuron driver *C380-GAL4 > UAS-CD8::GFP*; **(b)** Postsynaptic body wall muscle driver *C57-GAL4 > UAS-CD8::GFP*. White arrowheads denote NMJs on muscle 6/7 of segment A3. On the muscles, the membrane-rich subsynaptic reticulum (SSR) surrounds the boutons. Thus, when the membrane-tethered CD8::GFP was expressed in the muscles by *C57-GAL4* in **(b)**, strong green signals were observed at the SSR in the shape of boutons, even though there was no CD8::GFP expression in the presynaptic boutons. Anti-GFP (in green). Anti-HRP (in red). Scale bar is 200 μm.

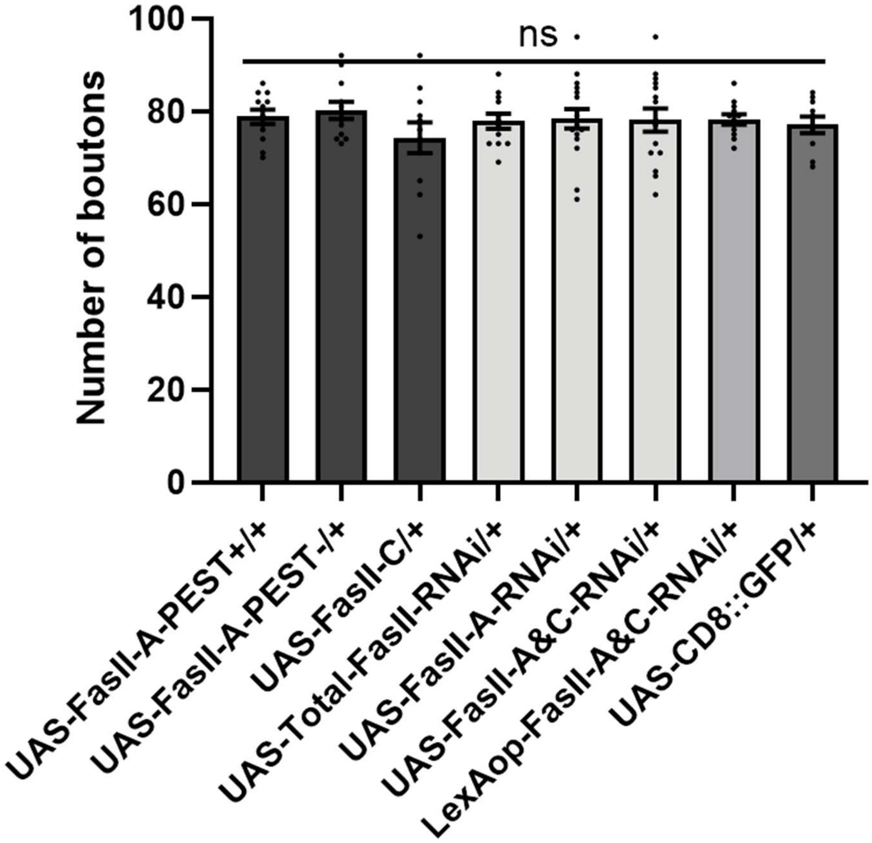

Supplementary Figure 21. No notable abnormalities were found in the bouton numbers of the genetic controls for the major UAS lines used in this study. Quantification of *Drosophila* NMJs of late 3rd instar larvae at muscles 6 and 7 of segment A3. n = 12, 12, 11, 12, 17, 15, 10, 12, where n is the number of NMJs analyzed. Each larva is defined as a biological replicate, and no more than two NMJs were analyzed per larva. One-way ANOVA with Tukey post-hoc test was performed.

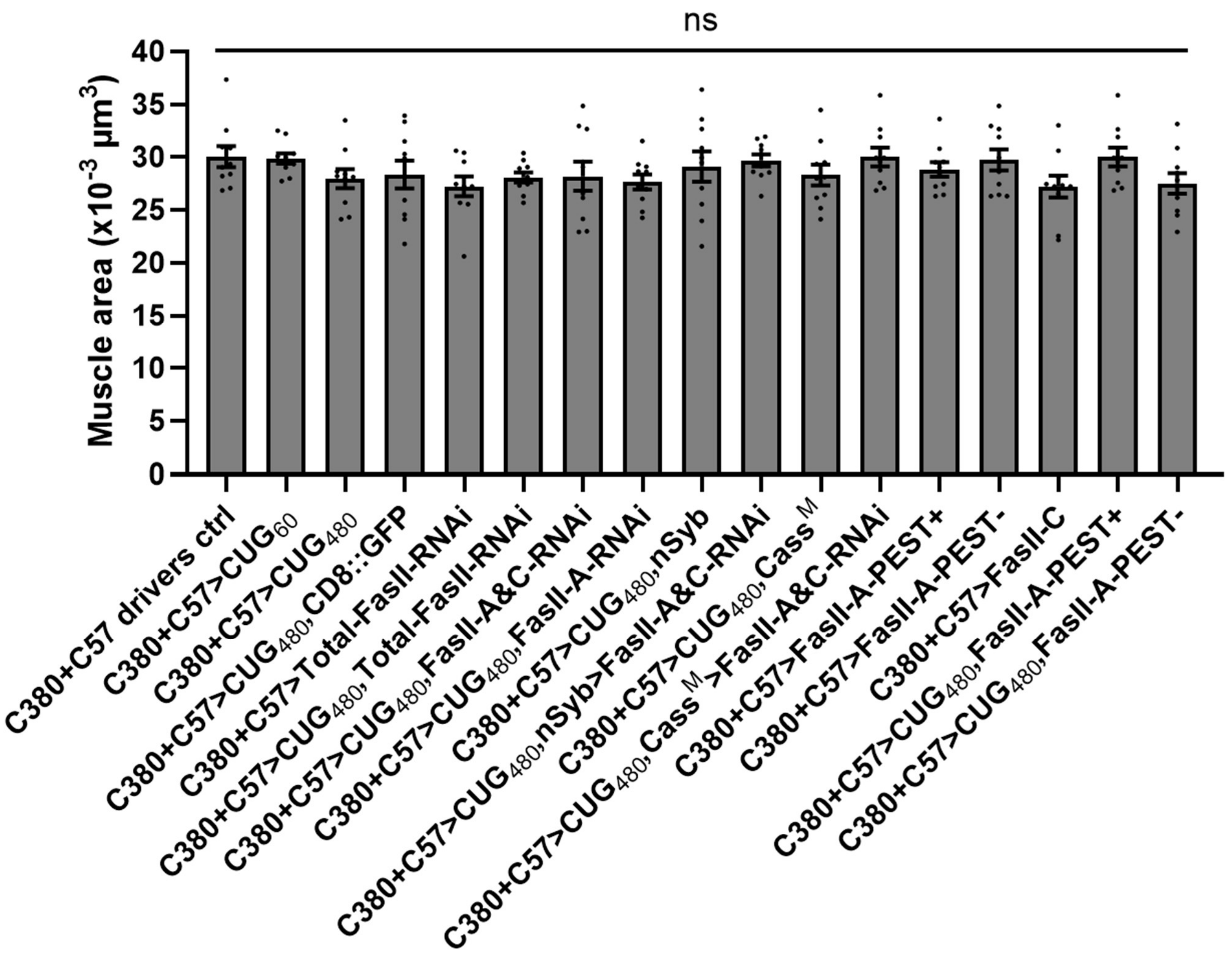

Supplementary Figure 22. No notable differences were found among the size of body wall muscles of the major genotypes used in this study. Quantification of muscle area (Muscle 6, Segment A3) for major genotypes used in this study. n = 10 for all genotypes, where n is the number of muscles analyzed. Each larva is defined as a biological replicate, and no more than two NMJs were analyzed per larva. One-way ANOVA with Tukey post-hoc test was performed.
