# Supplementary material for: Pre- and postsynaptic upregulation of FasII synergistically underlies neuropathological and behavioral phenotypes in a Drosophila model of myotonic dystrophy
Source: Nat Commun. 2025 Dec 18;17:1005. doi: 10.1038/s41467-025-67738-w (PMC12847815; doi:10.1038/s41467-025-67738-w)
Supplement: Supplementary file 2 — Reporting Summary [file 41467_2025_67738_MOESM2_ESM.pdf]

nature portfolio

Corresponding author(s): Ho Yin Edwin CHAN

Last updated by author(s): Dec 4, 2025

Reporting Summary

Nature Portfolio wishes to improve the reproducibility of the work that we publish. This form provides structure for consistency and transparency in reporting. For further information on Nature Portfolio policies, see our Editorial Policies and the Editorial Policy Checklist.

Statistics

For all statistical analyses, confirm that the following items are present in the figure legend, table legend, main text, or Methods section.

n/a	Confirmed	
☐	☒	The exact sample size (*n*) for each experimental group/condition, given as a discrete number and unit of measurement
☐	☒	A statement on whether measurements were taken from distinct samples or whether the same sample was measured repeatedly
☐	☒	The statistical test(s) used AND whether they are one- or two-sided<br>*Only common tests should be described solely by name; describe more complex techniques in the Methods section.*
☐	☒	A description of all covariates tested
☐	☒	A description of any assumptions or corrections, such as tests of normality and adjustment for multiple comparisons
☐	☒	A full description of the statistical parameters including central tendency (e.g. means) or other basic estimates (e.g. regression coefficient) AND variation (e.g. standard deviation) or associated estimates of uncertainty (e.g. confidence intervals)
☐	☒	For null hypothesis testing, the test statistic (e.g. *F*, *t*, *r*) with confidence intervals, effect sizes, degrees of freedom and *P* value noted<br>*Give P values as exact values whenever suitable.*
☒	☐	For Bayesian analysis, information on the choice of priors and Markov chain Monte Carlo settings
☒	☐	For hierarchical and complex designs, identification of the appropriate level for tests and full reporting of outcomes
☒	☐	Estimates of effect sizes (e.g. Cohen's *d*, Pearson's *r*), indicating how they were calculated

Our web collection on statistics for biologists contains articles on many of the points above.

Software and code

Policy information about availability of computer code

Data collection	For electrophoresis and slot blot analysis, image acquisition and quantification were conducted with Bio-Rad ChemiDoc MP Imaging System and software (Image Lab 6.0.1).<br>For electrophysiology, data were acquired using an Axoclamp 900A amplifier, Digidata 1440A acquisition system and pClamp10.7 (Molecular Devices) software.<br>For creation of figures, Adobe Photoshop CS5 Extended Version 12.0 was used to create TIF files with layers.
Data analysis	Slot blot images were analyzed with Image Lab software (Bio-Rad Laboratories, Hercules, CA, USA).<br>Densitometric analysis of slot blots and semiquantitative RT-PCR was performed using ImageJ software (Research Services Branch, National Institute of Mental Health).<br>For electrophysiology, data were analyzed using MiniAnalysis (Synaptosoft) and the Clampfit (Molecular Devices) programs.<br>Statistical analyses were performed with GraphPad Prism, versions 9 (GraphPad Software, Inc).

For manuscripts utilizing custom algorithms or software that are central to the research but not yet described in published literature, software must be made available to editors and reviewers. We strongly encourage code deposition in a community repository (e.g. GitHub). See the Nature Portfolio guidelines for submitting code & software for further information.

Data

Policy information about availability of data

All manuscripts must include a data availability statement. This statement should provide the following information, where applicable:

- Accession codes, unique identifiers, or web links for publicly available datasets
- A description of any restrictions on data availability
- For clinical datasets or third party data, please ensure that the statement adheres to our policy

All data supporting the findings of this study are presented within the main manuscript and Supplementary Information. All the data generated in this study are provided in the Source Data file.

Research involving human participants, their data, or biological material

Policy information about studies with human participants or human data. See also policy information about sex, gender (identity/presentation), and sexual orientation and race, ethnicity and racism.

Reporting on sex and gender	For frontal cortex samples:<br>DM1 patients: 3 males, 4 females<br>Non-DM controls: 4 males, 1 female<br><br>For transdifferentiated myoblasts donors:<br>DM1 donor: 1 female<br>Non-DM controls: 1 male
Reporting on race, ethnicity, or other socially relevant groupings	N/A
Population characteristics	For frontal cortex samples:<br>DM1 patients:<br>Genotype/diagnosis: DM1, CTG repeat expansion detected by Souther blot or PCR amplification.<br>Age: Mean, 64.3; SD, 5.1<br>Age of onset: mean, 46,0; SD, 12.5 (all patients presented adult onset clinical forms)<br>Cause of death: pneumonia or heart failure.<br><br>Non-DM controls:<br>Clinical diagnosis: No clinical diagnosis (2 individuals), Charcot-Marie Tooth Disease (1 individual), Rheumathoide arthrisis (1 individual), Limb-Girdle muscular dystrophy (1 individual).<br>Genotype: Absence of DM1 or DM2 repeat expansion.<br>Age: Mean, 68.6; SD, 10.4<br>Cause of death: pneumonia or heart failure.<br><br>For transdifferentiated myoblasts donors:<br>DM1 donor: 11-year-old female donor with DM1 (1,300 CTG repeats).<br>Non-DM control donor: 25-year-old healthy male.
Recruitment	For frontal cortex samples, post-mortem brain tissue was collected from adult DM1 patients with confirmed genetic diagnosis (Southern blot detection or PCR amplification of expanded CTG trinucleotide repeat expansion). Non-DM controls were recruited to match the age and sex of DM1 patients, whenever possible.<br>Individuals were recruited at Asahikawa Medical Center (Japan) and Okayama University (Japan).<br><br>For the transdifferentiated myoblasts, samples were obtained from the Institute of Myology, Paris. Skin biopsies were obtained from donors.
Ethics oversight	All experiments using human samples were approved by the local Ethics Committees of the host institutions where brain samples were collected: Asahikawa Medical Center (Japan) and Okayama University (Japan). Written informed consent of specimen use for research was obtained from all patients.<br><br>All muscle biopsies of the transdifferentiated myoblasts were taken after informed consent by patients and approval by the Experimentation Ethics Committee of the University Hospital La Fe (Valencia, Spain; authorization number: 2014/0799)

Note that full information on the approval of the study protocol must also be provided in the manuscript.

Field-specific reporting

Please select the one below that is the best fit for your research. If you are not sure, read the appropriate sections before making your selection.

[x] Life sciences [ ] Behavioural & social sciences [ ] Ecological, evolutionary & environmental sciences

Life sciences study design

All studies must disclose on these points even when the disclosure is negative.

Sample size	No sample size calculation was performed for this study. Sample size was chosen based on previous experience and historical data collected in the laboratory for each individual assay. For NMJ analysis, a minimum of 10 NMJs were used. A maximum of 2 NMJs are used per larva. For larval crawling behavioral analysis, a minimum of 20 larvae (n of 20) were used. For each larva, 2 recordings were performed, and the average distanced crawled was calculated, which was represented by n of 1. For semiquantitative RT-PCR and slot blot experiments, a minimum of 3 biological replicates, up to a maximum of 7 were studied, to account for variability between samples.
Data exclusions	Out of the five independent astrocyte cultures transfected with Mbnl1/Mbnl2 siRNA or scrambled siRNA, one did not grow. Non-transfected control cultures derived from the same embryo did not grow either. This replicate was excluded from the analysis.<br>No additional data were excluded from the experiments and analyses presented.
Replication	All experiments were reproduced to reliably support conclusions stated in the manuscript. For NMJ analysis and behavioral analysis, each cross was repeated at least twice to ensure the results are repeatable.
Randomization	Our study is not subject to randomization since it does not involve allocation of participants or samples into experimental groups.
Blinding	Investigators who conduct the experiments were blinded to group allocation during data collection and analysis. When handling animals, blinding was not always possible, because experienced investigators may be able to distinguish transgenic animals from wild-type controls, given the difference in body weight. The investigator was blind to the genotype being tested, whenever possible. The investigator was blind to the genotype of astrocytes acutely isolated from adult mice.

Reporting for specific materials, systems and methods

We require information from authors about some types of materials, experimental systems and methods used in many studies. Here, indicate whether each material, system or method listed is relevant to your study. If you are not sure if a list item applies to your research, read the appropriate section before selecting a response.

Materials & experimental systems

n/a	Involved in the study	
☐	☒	Antibodies
☒	☐	Eukaryotic cell lines
☒	☐	Palaeontology and archaeology
☐	☒	Animals and other organisms
☒	☐	Clinical data
☒	☐	Dual use research of concern
☒	☐	Plants

Methods

n/a	Involved in the study	
☒	☐	ChIP-seq
☒	☐	Flow cytometry
☒	☐	MRI-based neuroimaging

Antibodies

Antibodies used	Alexa Fluor 488 Goat Anti-Horseradish Peroxidase (RRID: AB_2338965) (Jackson Lab).<br>Alexa Fluor 647 Goat Anti-Horseradish Peroxidase (RRID: AB_2338967) (Jackson Lab).<br>Mouse Anti-discs large (DLG) 4F3 (DSHB - Developmental Studies Hybridoma Bank).<br>Mouse Anti-Fasciclin II (Fas2) 1D4 (DSHB - Developmental Studies Hybridoma Bank).<br>Mouse Anti-Fasciclin II (Fas2) 34B3 (DSHB - Developmental Studies Hybridoma Bank).<br>Goat Anti-Mouse IgG H&L (Alexa Fluor 488) (Abcam, ab150113).<br>Goat Anti-Mouse IgG H&L (Alexa Fluor 594) (Abcam, ab150116).<br>Rabbit Anti-NCAM1 antibody (Abcam, ab154566).<br>Rabbit Anti-beta Tubulin antibody - Loading Control (Abcam, ab6046).<br>β-tubulin Antibody (2G7D4), mAb, Mouse (Genscript, cat. no. A01717).
Validation	Alexa Fluor 488 Goat Anti-HRP (RRID: AB_2338965) (Jackson Lab). 44 references on the manufacturer's website.<br>Alexa Fluor 647 Goat Anti-HRP (RRID: AB_2338967) (Jackson Lab). 122 references on the manufacturer's website.<br>Mouse Anti-discs large (DLG) 4F3 (DSHB). 94 references on the manufacturer's website.<br>Mouse Anti-Fasciclin II (Fas2) 1D4 (DSHB). 77 references on the manufacturer's website.<br>Mouse Anti-Fasciclin II (Fas2) 34B3 (DSHB). 1 reference on the manufacturer's website.<br>Goat Anti-Mouse IgG H&L (Alexa Fluor 488) (Abcam, ab150113). 1170 references on the manufacturer's website.<br>Goat Anti-Mouse IgG H&L (Alexa Fluor 594) (Abcam, ab150116). 421 references on the manufacturer's website.<br>Rabbit Anti-NCAM1 antibody (Abcam, ab154566). Tested reactivity: Mouse, Rat. Predicted to work with: Human, Chicken, Cow, Cat, Dog. Tested applications: WB. 1 reference on the manufacturer's website.<br>Rabbit Anti-beta Tubulin antibody - Loading Control (Abcam, ab6046). Tested reactivity: Mouse, Rat, Human. Tested applications: WB, ICC/IF, IHC-P, IP. 1124 references on the manufacturer's website.

	β-tubulin Antibody (2G7D4), mAb, Mouse (Genscript, cat. no. A01717). Tested reactivity: Human, Monkey, Goat, Bovine, Swine, Rabbit, Rat, Mouse, Chicken and Fish. Tested applications: WB, Flow cytometry, IHC. 2 references on the manufacturer’s website.

Animals and other research organisms

Policy information about studies involving animals; ARRIVE guidelines recommended for reporting animal research, and Sex and Gender in Research

Laboratory animals	Species: Mus musculus.<br>Strains: C57BL/6 and DMSXL transgenic mice (>99 C57BL/6 background), carry a 45 kb fragment of human genomic DNA containing the DMPK gene with an expanded 1,300 CTG repeat.<br>Age of animals for tissue collection: Postnatal day P16-P21.<br><br>Housing Conditions:<br>Mice were kept in ventilated racks (Tecniplast GM500 cages, Buguggiate, Italy) under standard conditions, including a 12-hour light/dark cycle (lights on from 7:00 to 19:00), with room temperatures maintained between 22 °C and 24 °C and relative humidity ranging from 20% to 42%. Cage changes occurred every two weeks, with no changes made during the week of behavioral testing. The animals had access to water through non-automated bottles and were provided with ad libitum feeding of Safe A04 chow.
Wild animals	The study did not involve wild animals.
Reporting on sex	2 female and 3 male mice aged between 16 – 21 days were used for each genotype analyzed.
Field-collected samples	The study did not involve samples collected from the field.
Ethics oversight	Animal ethics was approved by Ministry of Higher Education, Research and Innovation (Paris). Authorization for animal experimentation number #23473. Animal facility approval number B751320.

Note that full information on the approval of the study protocol must also be provided in the manuscript.

Plants

Seed stocks	N/A
Novel plant genotypes	N/A
Authentication	N/A
